# Evaluation of the Hindi version of the London Measure of Unplanned Pregnancy among pregnant and postnatal women in urban India

**DOI:** 10.1186/s12884-021-04075-y

**Published:** 2021-09-04

**Authors:** Sushmita Das, Jennifer Hall, Geraldine Barrett, David Osrin, Shaili Kapadia, Anuja Jayaraman

**Affiliations:** 1 Society for Nutrition, Education and Health Action, Behind Bldg. No. 11, BMC Colony, Shastri Nagar, Santa Cruz (W), 400 054 Mumbai, India; 2 UCL Elizabeth Garrett Anderson Institute for Women‘s Health, University College London, Medical School Building, 74 Huntley Street, WC1E 6AU London, UK; 3 UCL Institute for Global Health, 30 Guilford Street, WC1N 1EH London, UK; 4 Columbia University Mailman School of Public Health, 722 W 168th St, NY 10032 New York, USA

**Keywords:** Pregnancy intention, Psychometric, Validation, India, Measure, Unplanned

## Abstract

**Background:**

Valid and reliable measures such as London Measure of Unplanned Pregnancy (LMUP) are imperative for understanding fertility-related behaviors and estimating unintended pregnancy. The aim of this study was to validate the LMUP in the Hindi language for a wider reach in India.

**Methods:**

An interviewer administered version of the LMUP was translated and pretested in Hindi. The LMUP was field tested with married women in the reproductive age group across forty informal settlements in Mumbai in the post intervention census of a cluster randomized control trial to improve the health of women and children. Analyses involved the full sample and sub-groups according to time-from-conception. Reliability (internal consistency) was assessed using Cronbach’s alpha, inter-item correlations, and item-rest correlations. Construct validity was assessed by hypothesis testing and confirmatory factor analysis.

**Results:**

4991 women were included in the study (1180 were pregnant, 2126 in their first- and 1685 in their second postnatal year). LMUP item completion rates were 100 % and the full range of LMUP scores was captured. Reliability: the scale was internally consistent (Cronbach’s α = 0.84), inter-item correlations were positive, and item-rest correlations were above 0.2 for all items except item six (0.07). Construct validity: hypotheses were met, and confirmatory factor analysis showed that a one-factor model was a good fit for the data, confirming unidimensional measurement. The sub-group analysis (by pregnant, first-, and second postnatal year) showed that the psychometric properties of the LMUP were similar across the groups. In terms of LMUP scores, the women in the postnatal groups were very slightly, but significantly, more likely to have an LMUP score of 10 + compared to pregnant women; the difference between the first and second postnatal year was not significant.

**Conclusions:**

The Hindi LMUP is valid and reliable measure of pregnancy intention that may be used in India.

**Trial Registration:**

This study is registered with ISRCTN, number ISRCTN56183183, and Clinical Trials Registry of India, number CTRI/2012/09/003004.

## Background

Of the estimated 206 million pregnancies in low- and middle-income countries in 2017, 89 million (43 %) were unintended [1]. Unintended pregnancies are influenced by access to contraceptive methods [2, 3]. This access enables women and couples to have the number of children they want and to time births as they desire. Recent estimates suggest that 214 million women were not using any modern method of contraception irrespective of their intention to avoid any future pregnancy. Satisfying these unmet needs would have prevented 67 million unintended pregnancies, 36 million induced abortions, and 23 million unplanned births in 2017 [1].

The rate of unintended pregnancy in a population is a critical reproductive health measure [4]. Women with unintended pregnancies are at higher risk of developing complications such as high blood pressure, anemia, and gestational diabetes [5, 6]. These conditions, if not managed, can lead to severe obstetric complications like hemorrhage, pre-eclampsia, pre-term birth, and maternal mortality [7–9]. The risks are amplified because women with unintended pregnancies are less likely to seek early prenatal care [10, 11]. Children born of unintended pregnancies are more likely to have low birth weight, poor nutritional status, morbidity, and mortality [12–15]. Preventing unintended pregnancies can therefore lead to substantial improvement in maternal and child health outcomes [2, 13].

Valid, reliable, and agreed measures of unintended pregnancy are imperative for understanding fertility-related behaviors, estimating unmet need for contraception, and designing family planning programs that prevent unintended pregnancy [16]. A pregnancy is most commonly defined as unintended if it is either unwanted (occurred when no children or no more children were desired: sometimes termed “number failures”), or if conception is mistimed [17]. Estimates are derived from the Demographic and Health Surveys (DHS), which measure pregnancy intention by asking, “When you got pregnant, did you want to get pregnant at that time?” and “Did you want to have the baby later on or did you not want any (more) children?” While these questions provide fundamental data, they categorize pregnancy intention into dichotomous categories of “planned” or “unplanned”. Previous studies have shown that this categorization can be limiting and may not capture the complexity of intention and decision making in pregnancy [18, 19].

The London Measure of Unplanned Pregnancy (LMUP) is a six-item questionnaire which identifies intention of a current or recent pregnancy regardless of its outcome: birth, abortion or miscarriage [20]. It was developed in the United Kingdom (UK) and was originally created to be self-administered. The LMUP includes responses to questions on behavior (contraception, pre-conception preparation), stance (expressed intentions, desire for a baby) and context (timing, discussion with partner). Each item has three response categories (0, 1, or 2) and the responses are summed to derive a score between zero and twelve, where a higher score represents greater pregnancy intention. The scale avoids dichotomization into planned or unplanned pregnancy and instead allows a woman to express ambivalence and inconsistency [21]. LMUP scores of 0–3 describe pregnancy as unplanned, 4–9 as ambivalent, and 10–12 as planned [22] [23].

The LMUP is a useful tool for understanding and measuring pregnancy intention across a wide range of settings. Beyond the original work in the UK, it has been translated and validated for use in Saudi Arabia (Arabic), Malawi (Chichewa), Belgium (Dutch/Flemish), Iran (Persian), Brazil (Portuguese), Pakistan (Urdu), the United States of America (English and Spanish), India (Tamil and Kannada), Australia (English), Uganda (Luganda, Acholi, Lugisu, Runyakole), Sierra Leone (Krio), and Sri Lanka (Sinhala) [21, 23–33]. The objective of this study was to validate the LMUP for use in Hindi language for a wider reach in India.

## Methods

### Setting

India accounted for 48.1 million pregnancies in 2015. Almost half (48 %) were unintended and one third resulted in abortions [34]. Unintended pregnancies continue to compromise India’s maternal mortality rate (174 maternal deaths per 100,000 live births) by inessentially exposing women to pregnancy-related complications [35].

Mumbai, capital of the western state of Maharashtra and the largest metropolis in India, reported 13.7 % unmet need for family planning among currently married women aged 15–49 years [36]. The Municipal Corporation of Greater Mumbai is responsible for administration of the mega-city in three zones: city, central and western. The study was conducted in two of the 24 municipal wards with the city’s lowest Human Development Measures in terms of total literacy rate, proportion of slum population and marginal workers, and infant mortality rate (per 1000 live births) [37]. M East and L wards have Human Development Measures of 0.05 and 0.29, associated large migrant populations, poor education and health facilities, low and insecure levels of livelihood activity, and large-scale unauthorized housing (slums or informal settlements) [37].

The Society for Nutrition, Education and Health Action (SNEHA) is a non-profit organization working to improve the health and nutrition of women and children in informal settlements in Mumbai. In 2011, SNEHA conducted a cluster randomized control trial of an integrated model of community resource centers for improved women and child health outcomes in 40 areas of M East and L wards. We had chosen two wards on the basis of poorer human development index ranking and a high proportion of slum settlements. The areas within the wards were chosen based on their vulnerability status. Each area consisted of approximately 600 households. Some encompassed entire informal settlement areas and others were sections of larger geographical areas. All households in an area were included in the program intervention. The resource centers provided community-level services for health, nutrition, and domestic violence against women and children. The methods and results of the study are explained in detail elsewhere [38]. Data were collected from February 2014 to September 2015, in a census after the trial intervention, covering all households with married women in the reproductive age group.

### Translation and Pilot Testing

The LMUP was originally developed in English and was self-administered. Given low education levels in the study population, interviewer administration of the LMUP was considered a feasible alternative to self-administration, as has been done elsewhere [21, 24, 26, 29, 30, 32].

To adapt it to the local context, minor modifications were made to the original list of pre-pregnancy health behaviors (item six). We amended the “stopped or cut down smoking” response option to also include stopping or cutting down consumption of betel leaf, gutka (chewing tobacco) and beedi (hand rolled cigarettes). Cultural adaptation of item six is well established [21, 24, 27, 32, 33]. The English LMUP questions were translated by the research coordinator who was bilingual in both English and Hindi. The accuracy of the translation was confirmed by back-translating the questions. This was carried out by a research consultant who was fluent in Hindi and English and had over five years’ experience in qualitative research. There were no major changes in the back-translation except in one statement. Item four asked about the woman’s desire to have a baby and one of the answer choices, “I had mixed feelings about having a baby,” was edited to be clearer. Following back-translation, the LMUP was pilot tested with 15 women with similar socio-demographic characteristics to the study population. The objective was to ensure women understood the questions being asked and the purpose of collecting the LMUP data. A minor modification was made after pilot testing in terms of rewording the items to refer clearly to a husband rather than a partner because the word was confusing to respondents.

### Data Collection

The census was conducted by two teams of six investigators and one supervisor. Each team was responsible for data collection in 20 areas. Investigators visited homes in their areas up to three times to arrange interviews with married women in the reproductive age group in each household. During the meeting, investigators explained the purpose of the study and assured participants of the confidentiality of the data to be collected. Participants were then asked for written consent to interview. Questionnaires included modules on household and maternal characteristics. At the household level, information was collected on home ownership, housing construction, drinking water source, toilet facility, and household assets. Demographic information included the woman’s age, educational attainment, current employment status, and religion. Maternal history and the six LMUP questions were part of the maternal questionnaire. The LMUP was administered to women who were pregnant at the time of census or had a child under two years of age. Data were collected on smartphones running Open Data Kit (ODK: Seattle, WA, USA) in Google Android (versions 3.0-4.4). The system included validation constraints and automatic skips. Of the total interviews, 5 % were observed by a supervisor. Data were checked after download by the data manager for errors in key fields.

### Statistical analysis

The analytical strategy was based on Classical Test Theory which underpinned the development of the LMUP and has been employed by subsequent evaluations such as that of Hall et al. [20, 21]. This strategy included assessment of (1) acceptability and targeting, (2) reliability, and (3) validity. In this study it was also possible for us to carry out an analysis of the measurement properties of the Hindi LMUP across three groups of women according to their time from conception. The analysis was conducted in Stata/IC 15 (StataCorp, College Station, TX).

#### Acceptability and targeting

The acceptability of the LMUP was initially examined through the pilot interviews and further examined by assessing missing data rates. Lower levels of missing data give an indication of greater acceptability [39]. The distribution of total LMUP scores was checked to examine whether the full range of scores had been captured as an indication of the targeting of the measure. The proportion of women who selected each item response option (item endorsement) was examined to provide information about the discrimination of the item.

#### Reliability

To evaluate reliability (internal consistency), we calculated Cronbach’s α statistic using the standard cut-off point of 0.7 [40]. All item-rest correlations were assessed for positive values and a minimum correlation of 0.20 was considered acceptable [41]. We did not assess the reliability of the Hindi LMUP in terms of its stability (test-retest reliability) as we did not carry out repeat testing of the LMUP within the study.

#### Validity

We assessed the construct validity of the Hindi LMUP in several ways. First, we used hypothesis testing with known groups. Hypotheses were developed based on SNEHA’s program implementation experience, literature on pregnancy intention, and previous LMUP validations [21, 32]. The two hypotheses were [1] that women aged 30 years or less would be more likely to have higher LMUP scores, and [2] that women with four or more children would be more likely to have a lower LMUP score. We used the Kruskall Wallis test for inference. If a construct validity hypothesis is not met, the measure is failing to detect a known difference. Second, in keeping with recent standards of assessment of structural validity [42] (an aspect of construct validity), Confirmatory Factor Analysis (CFA) was conducted to confirm the hypothesis that the six questions of the LMUP were measuring one underlying construct [43]. The one-factor LMUP model was considered a good fit to the data if the Comparative Fit Index (CFI) had a value higher than 0.95 and the standardized root mean squared residual (SRMR) had a value less than 0.08 [43]. To address the non-normal and asymmetric nature of the LMUP items and total score, the asymptotic distribution free (ADF) estimation method (a form of weighted least squares) was chosen for the CFA model. Given that in previous evaluations of the LMUP the structural validity of the scale was assessed using principal components analysis (PCA), we also present PCA findings here to allow direct comparison with previous evaluations. In these, the unidimensionality of the scale was confirmed if all items loaded onto one component with an Eigenvalue greater than 1. Prior to the PCA and CFA analysis of the overall dataset, we assessed the Kaiser-Meyer-Olkin (KMO) measure of sampling adequacy (using the following interpretation: <0.70 mediocre or worse; 0.70–0.79 middling; 0.80–0.89 meritorious; =>0.90 marvelous) [44] and Bartlett’s test of sphericity (p < 0.05 indicating the dataset is suitable for data reduction) [45].

There is no existing “gold standard” for measuring pregnancy planning and it was therefore not possible to measure concurrent criterion validity of the LMUP using another measure.

#### Measurement properties of the LMUP according to time from conception

Given that the SNEHA dataset was large and included women who were pregnant through to the second postnatal year, we had the opportunity to carry out an analysis of the LMUP’s measurement properties according to time since conception by dividing women into three groups: pregnant, in the first postnatal year, and in the second postnatal year. Our expectation was that the measurement properties of the LMUP among the groups should be similar. We tested this as a hypothesis, assessing whether measurement properties of the LMUP (targeting, Cronbach’s alpha, item-rest correlations, PCA, and CFA) met the criteria for reliability, validity and targeting within each group independently.

Previous research has shown that women’s LMUP scores tend to increase slightly over time, suggesting greater intention. We assessed total LMUP scores by group to see if they showed consistency with the previous research by being higher in the groups further away from conception [46]. We compared LMUP scores across the three groups using the Kruskall Wallis test (to test for differences among the three distributions) and the Jonckheere-Terpstra test for ordered alternatives (to test whether there was a linear relationship between the ordered grouping variable and the distributions of LMUP scores). Given the difference in age and parity between the pregnant and postnatal groups, we conducted a multivariable logistic regression analysis using 9/10 as the cutpoint between unplanned and planned pregnancy to compare the LMUP scores of the pregnant and postnatal groups [22].

## Results

The census collected information from 16,236 women in the reproductive age group. The response rate for the census was 91 %; 7 % of women were not available at the time of data collection and 2 % refused to participate in the study. The LMUP, which was administered to women who were pregnant at the time of census or had a child under two years of age, was completed by 4991 women.

### Sample characteristics

Table 1 describes the socio-demographic and household characteristics of the respondents. The household information included home ownership status, housing construction type, electricity supply, drinking water source, toilet facility, and socioeconomic status. Most women (74 %) lived in permanent housing. Most households (78 %) accessed drinking water at a public or community tap stand and 83 % used a public or shared toilet facility. Mean age of women was 27 (SD 5.05, median 25, IQR 23–30) with number of children ranging from zero to 15 (mean 2.4, SD 1.7, median 2 IQR 1–3). At the time of survey, 23 % women were pregnant, 43 % were in their first postnatal year, and 34 % were in their second. 27 % of women had no formal schooling. Most women (96 %) were not working, 84 % identified as Muslim, and 99 % were either married or cohabitating with their partner.

**Table 1:** Socio-demographic characteristics of women completing LMUP

	Pregnant		1st postnatal year		2nd postnatal year		Total	
	n	**Percent**	n	**Percent**	n	**Percent**	n	**Percent**
**Women respondents**	1180	**(23)**	2126	**(43)**	1685	**(34)**	4991	**(100)**
**Household characteristics**								
**Home ownership**								
Own home	563	**(48)**	1082	**(51)**	906	**(54)**	2551	**(51)**
Rented home	617	**(52)**	1044	**(49)**	779	**(46)**	2440	**(49)**
**Housing construction**								
Temporary (kaccha)	318	**(27)**	579	**(27)**	425	**(25)**	1322	**(26)**
Robust (pucca)	862	**(73)**	1547	**(73)**	1260	**(75)**	3669	**(74)**
**Electric supply**								
Has electricity	1180	**(100)**	2125	**(100)**	1685	**(100)**	4990	**(100)**
**Drinking water source**								
Public tapstand	921	**(78)**	1675	**(79)**	1278	**(76)**	3874	**(78)**
Tap at home	259	**(22)**	451	**(21)**	407	**(24)**	1117	**(22)**
**Toilet facility**								
Public or shared	979	**(83)**	1785	**(84)**	1381	**(82)**	4145	**(83)**
Private	201	**(17)**	341	**(16)**	304	**(18)**	846	**(17)**
**Socio-economic status**								
Poorest tertile 1	394	**(34)**	755	**(36)**	598	**(35)**	1747	**(35)**
Tertile 2	405	**(34)**	663	**(31)**	567	**(34)**	1635	**(33)**
Least poor tertile 3	381	**(32)**	708	**(33)**	520	**(31)**	1609	**(32)**
**Women**								
**Age in years**								
16–19	62	**(5)**	45	**(2)**	27	**(2)**	134	**(3)**
20–24	632	**(54)**	1014	**(48)**	739	**(44)**	2385	**(48)**
25–29	347	**(29)**	754	**(35)**	597	**(35)**	1698	**(34)**
30+	139	**(12)**	313	**(15)**	322	**(19)**	774	**(15)**
**Education**								
No formal schooling	328	**(28)**	547	**(26)**	463	**(27)**	1338	**(27)**
Primary	55	**(5)**	101	**(5)**	79	**(5)**	235	**(5)**
Secondary	674	**(57)**	1274	**(60)**	989	**(59)**	2937	**(59)**
Higher	123	**(10)**	203	**(9)**	153	**(9)**	479	**(9)**
Missing	-	**-**	1	**(< 1)**	1	**(< 1)**	2	**(< 1)**
**Parity**								
None	335	**(29)**	-	**-**	-	**-**	335	**(7)**
1–3 children	699	**(59)**	1585	**(75)**	1254	**(74)**	3538	**(71)**
4 or more children	146	**(12)**	541	**(25)**	431	**(26)**	1118	**(22)**
**Occupation**								
Not working	1137	**(96)**	2049	**(96)**	1601	**(95)**	4787	**(96)**
Working	43	**(4)**	77	**(4)**	84	**(5)**	204	**(4)**
**Religion**								
Muslim	1003	**(85)**	1803	**(85)**	1394	**(83)**	4200	**(84)**
Hindu	175	**(15)**	318	**(15)**	285	**(17)**	778	**(16)**
Other	2	**(< 1)**	5	**(< 1)**	6	**(< 1)**	13	**(< 1)**
**Marital Status**								
Married or cohabitating	1176	**(100)**	2100	**(99)**	1644	**(98)**	4920	**(99)**
Widowed, divorced, or separated	4	**(< 1)**	26	**(1)**	41	**(2)**	71	**(1)**

### Acceptability and targeting

**Fig. 1:**
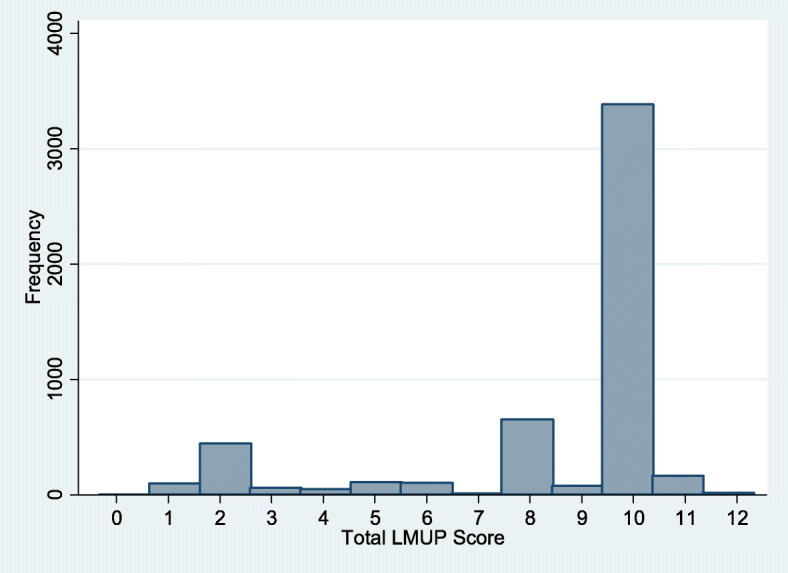
Frequencies of LMUP scores among women who were pregnant or within two years postpartum

There were no missing responses. The full range of LMUP scores was captured (zero to twelve) in the census (Fig. 1). Scores were not normally distributed and had a median of 10 (IQR 8–10) and a mean of 8.6 (SD 2.6). 11 % of women had a score of 0–3 (unplanned), 19 % scored 4–9 (ambivalent), and 70 % scored 10–12 (planned).

Table 2 illustrates women’s responses to individual LMUP questions. Items one (contraception) and six (preconception preparations) showed the least item discrimination. Most participants (94 %) were not using a method of contraception in the month they became pregnant (item one). When looking at preconception preparation (item six), almost all women (96 %) did not take any action to prepare for their pregnancy.

**Table 2:** Participant responses to LMUP questions

Scores	Total		Scores	Total	
	n	**Percent**		**n**	**Percent**
1. **Contraception**			4. **Desire**		
0	36	**(1)**	0	582	**(12)**
1	232	**(5)**	1	209	**(4)**
2	4723	**(94)**	2	4200	**(84)**
2. **Timing**			5. **Partner**		
0	543	**(11)**	0	1208	**(24)**
1	244	**(5)**	1	216	**(4)**
2	4204	**(84)**	2	3567	**(71)**
3. **Intention**			6. **Preparation**		
0	579	**(12)**	0	4778	**(96)**
1	205	**(4)**	1	194	**(4)**
2	4207	**(84)**	2	19	**(< 1)**

### Reliability

Cronbach’s α for the entire scale was 0.84. Inter-item correlations were all positive. Item-rest correlations were above 0.2 for all items except item six (0.07) (Table 3).

**Table 3:** Item-rest correlations, Confirmatory Factor Analysis and Principal Components Analysis loadings

Items	Item-rest correlations	Confirmatory factor Analysis: factor loadings	Component loadings (Eigenvalue = 3.5)
1. Contraception	0.25	0.28	0.36
2. Timing	0.91	0.98	0.97
3. Intention	0.91	0.99	0.97
4. Desire	0.91	0.99	0.97
5. Partner	0.60	0.62	0.72
6. Preparation	0.07	0.07	0.10

### Validity

**Fig. 2:**
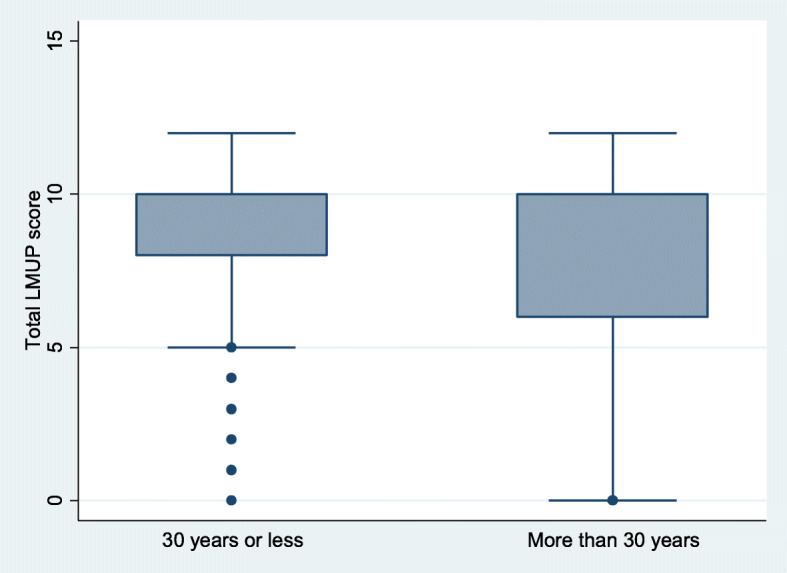
Box plot showing median and inter-quartile range of LMUP scores by women’s age group

**Fig. 3:**
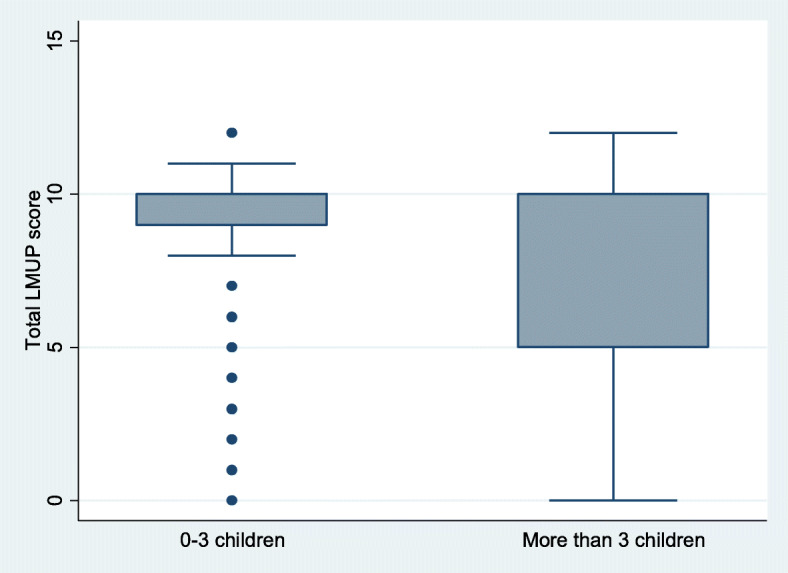
Box plot showing median and inter-quartile range of LMUP score by number of living children

Both construct validity hypotheses were confirmed. Women 30 years of age or above (p = 0.0001), and women with four or more children (p = 0.0001), were more likely to report their pregnancies as unintended (Figs. 2 and 3). The mean LMUP score for women under 30 years was higher (mean 8.8, SD 2.5, median 10, IQR 8–10) than for women above (mean 7.7, SD 3.2, median 10, IQR 6–10). Women with less than four children had a higher mean LMUP score (mean 8.9, SD 2.3, median 10, IQR 9–10) than women with more than three children (mean 7.4, SD 3.3, median 10, IQR 5–10).

Confirmatory factor analysis showed that a one-factor model was a good fit for the data (CFI 0.99, SRMR 0.02). Principal components analysis confirmed that the six items loaded onto one component with eigenvalue 3.5 (Table 3). The KMO was 0.85 and Bartlett’s test for sphericity *p* < 0.001.

### Measurement properties of the LMUP according to time from conception

#### Sample characteristics

We did not observe any differences in education level, employment status, or religion across the three groups. The only difference in household characteristics was in home ownership. More than half (59 %) of the pregnant women were in the age group 16–24 years. The proportion was lower for women in their first (50 %) and second (46 %) postnatal years. As expected, women in their first and second postnatal years had more children than pregnant women.

#### Acceptability and targeting

There were no missing responses. The full range of LMUP scores from zero to twelve was present in the groups of pregnant women and women in their first postnatal year. Women in their second postnatal year had a range of scores from one to twelve.

#### Reliability

Cronbach’s α was 0.86 for pregnant women, 0.83 for women in their first postnatal year, and 0.82 for women in their second. Inter-item correlations were positive in all three groups. Item-rest correlations were above 0.2 for items 1–5 in all three groups, except for item one which was borderline (0.19) in the second postnatal year. The item-rest correlation for item six was less than 0.2 in all three groups (Table 4).

**Table 4:** Item-rest correlations, Confirmatory Factor Analysis factor loadings and Principal Components Analysis loading by pregnancy and postnatal status

Items	Pregnant			1st postnatal year			2nd postnatal year		
	**Item-rest correlations**	**Confirmatory factor Analysis: factor loadings**	**Component loadings (Eigenvalue = 3.6)**	**Item-rest correlations**	**Confirmatory factor Analysis: factor loadings**	**Component loadings(Eigenvalue = 3.4)**	**Item-rest correlations**	**Confirmatory factor Analysis: factor loadings**	**Component loadings (Eigenvalue = 3.4)**
1) Contraception	0.29	0.31	0.40	0.26	0.28	0.36	0.19	0.21	0.28
2) Timing	0.92	0.99	0.97	0.90	0.99	0.97	0.89	0.98	0.96
3) Intention	0.92	0.99	0.97	0.90	0.99	0.97	0.90	0.99	0.97
4) Desire	0.93	0.99	0.98	0.91	0.99	0.97	0.90	0.99	0.97
5) Partner	0.65	0.67	0.77	0.58	0.60	0.71	0.58	0.60	0.71
6) Preparation	0.09	0.08	0.13	0.08	0.08	0.11	0.05	0.05	0.07

#### Validity

Hypothesis testing for each of the three groups confirmed that older women (> 30 years) and women with more children (> 3 children) were more likely to report their pregnancies as unintended. Results were significant for both older women and women with more children (> 3 children) (p = 0.0001 for each of the three groups). The mean LMUP score for pregnant women with less than four children (mean 8.6, SD 2.7, median 8, IQR 8–10) was higher than for women with four or more children (mean 6.2, SD 3.7, median 8, IQR 2–10). Similar results were observed in the first postnatal (mean 9, SD 2.1, median 10, IQR 10–10 for < = 3 children vs. mean 7.4, SD 3.3, median 10, IQR 5–10 for > 3 children) and second postnatal group (mean 9, SD 2.1, median 10, IQR 10–10 for < = 3 children vs. mean 7.4, SD 3.3, median 10, IQR 5–10 for > 3 children). Pregnant women aged < = 30 years had a higher mean LMUP score (mean 8.4, SD 2.8, median 10, IQR 8–10) than older pregnant women (mean 7.3, SD 3.4, median 10, IQR 3–10). Observations were similar for the first postnatal (mean 8.8, SD 2.4, median 10, IQR 8–10 for women < = 30 years vs. mean 7.7, SD 3.2, median 10, IQR 6–10 for women > 30 years) and second postnatal groups (mean 9, SD 2.2, median 10, IQR 10–10 for women < = 30 years vs. mean 7.8, SD 3, median 10, IQR 6–10 for > 30 years).

CFA confirmed the single factor LMUP model as a good fit for the data for every group: pregnant women – CFI 1.0, SRMR 0.02; first postnatal year – CFI 1.0, SRMR 0.01; second postnatal year – CFI 0.99, SRMR 0.04). Principal components analysis showed that all six items loaded onto one component in each group with eigenvalue of 3.6 for pregnant women, 3.4 for women in their first postnatal year, and 3.4 for women in their second. Item six had the lowest component loading in all three groups.

#### LMUP scores across groups

**Fig. 4:**
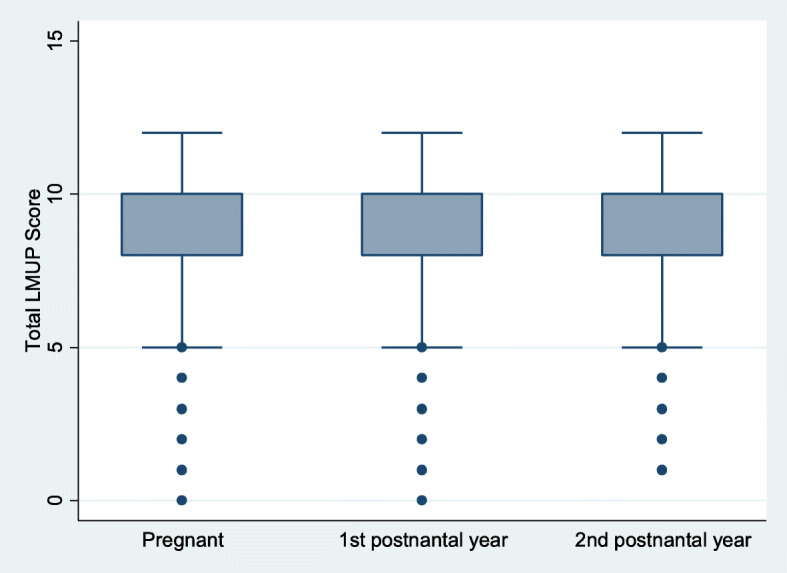
Box plot showing median and inter-quartile range of LMUP score by pregnancy and postnatal status

The median score was 10 (IQR 8–10) for each of the groups. Minor variation was observed in the mean scores of pregnant (8.3, SD 2.9), first (8.6, SD 2.6) and second (8.8, SD 2.4) year postnatal groups (Kruskall Wallis *p* = 0.002, Jonckheere-Terpstra test *p* = 0.0002) (Fig. 4).

Multivariable logistic regression showed that the differences in LMUP scores remained after adjusting for parity and age. After controlling for age and parity, women in their first postnatal year (AOR 1.36 (95 %CI 1.16–1.59)) and second postnatal year (AOR 1.63 (95 %CI 1.37–1.92)) were more likely to report their pregnancy as planned than were pregnant women, although the difference between the first and second postnatal year was not significant.

## Discussion

Our results indicate that the Hindi version of LMUP is reliable and valid in terms of acceptability, targeting, internal consistency and construct validity according to internationally accepted psychometric criteria, and therefore can be used for measuring pregnancy intention in Hindi speakers in India.

Our large population sample included women who were pregnant through to the second postnatal year and allowed us the opportunity of examining the psychometric properties of the Hindi LMUP by group according to time since conception: pregnant women, women in their first postnatal year, and women in their second. In the first such analysis, the LMUP performed in the same way in each group. Using the same three groups, we were also able to assess the LMUP score according to time from conception, testing the hypothesis that LMUP scores will be higher in groups further from the time of conception. This hypothesis was based on previous longitudinal work which showed that LMUP scores tend to increase the further the time from pregnancy [46]. In this analysis we found that LMUP scores were slightly, but significantly, higher in the postnatal groups than in the pregnant group. The differences in means across the groups were less than one LMUP point and would have relatively little impact in terms of understanding prevalence estimates of pregnancy intention. However, these data do reinforce the recommendation that it is best to measure pregnancy intention as close to conception as possible, ideally in pregnancy or at least at the first postnatal opportunity.

Of all the items in the LMUP, item six on preconception preparation was the least discriminating, and contributed least to the scale in this analysis. However, item six showed no evidence of being misunderstood and the LMUP was still internally consistent and unidimensional with item six. In India, although safe motherhood and newborn care are integral components of national health programs, the critical constituent of preconception health remains neglected. Good pre-pregnancy care is uncommon even in high-income countries and only a small proportion of women follow the recommended behaviors [47–50]. Given the context, it is not surprising that a small number of women in our study reported any preparation for improving preconception health. This may change over time with growing international efforts to improve preconception health. Given that item six does not harm the measure (and has the potential to reflect any future increase in preconception activities) we recommend retaining the item as use of the complete six-item measure facilitates international comparisons of the circumstances of women’s pregnancies. Of course, if the pattern of potential and actual preconception activities in India should change in future, the item could be adapted further to ensure its relevance to Hindi-speaking women.

The LMUP has been validated previously in two Indian languages, Tamil and Kannada. Minor modifications were made to the original version to suit the local context [32]. Although item two enquired whether a woman felt that her pregnancy came at the right time, not quite right time, or the wrong time, ‘time’ was interpreted by the respondents in Tamil and Kannada versions as a certain auspicious period in a week or month. Accordingly, the question was modified in both versions to ask if the woman wanted the pregnancy then, sooner, later, or not at all. Pre-pregnancy health behaviors listed in item six were also modified to suit the local context in Tamil and Kannada. In our study, we modified the list of preconception behaviors to include stopping or cutting down on the chewing tobacco, beedi and betel leaves, commonly consumed by women. We did not need to amend item two (timing) as women’s understanding of the Hindi translation was consistent with the original LMUP. No additional amendments were made to the original LMUP.

The strengths of the study were its large sample size and the opportunity to evaluate the LMUP in three subgroups of women. Limitations included a lack of scope for test-retest reliability assessment as women were interviewed only once and were not followed up over time. The study did not include women who reported spontaneous or medical abortion, or women who were unmarried.

## Conclusions

Our analysis suggests that the Hindi LMUP is a valid and reliable measure of pregnancy intention for use in India. As a psychometrically-validated measure of pregnancy intention, it is more robust than the analogous DHS module. At the same time, we recognize the need to substantiate the findings in other populations of Hindi-speaking women. Our study validated the LMUP in married women in urban informal settlements in a megacity and may not be representative of all women in India. We recommend further testing of the LMUP among women who are unmarried, live in rural areas, and belong to higher socio-economic groups.

## Data Availability

The datasets generated and/or analysed during the current study are available in the Open Science Framework repository, https://osf.io/kwzgx. The Hindi LMUP is available from: www.lmup.org.uk/docs/LMUP_Hindi.pdf.

## Abbreviations

LMUP: London Measure of Unplanned Pregnancy
DHS: Demographic and Health Surveys
SNEHA: Society for Nutrition, Education and Health Action
ODK: Open Data Kit
CFA: Confirmatory Factor Analysis
CFI: Comparative Fit Index
SRMR: Standardized root mean squared residual
PCA: Principal component analysis
